# Reduced Flow-Mediated Dilatation Is Not Related to COVID-19 Severity Three Months after Hospitalization for SARS-CoV-2 Infection

**DOI:** 10.3390/jcm10061318

**Published:** 2021-03-23

**Authors:** Marianne Riou, Walid Oulehri, Cedric Momas, Olivier Rouyer, Fabienne Lebourg, Alain Meyer, Irina Enache, Cristina Pistea, Anne Charloux, Christophe Marcot, Frederic de Blay, Olivier Collange, Michel Mertes, Emmanuel Andrès, Samy Talha, Bernard Geny

**Affiliations:** 1 Team 3072 “Mitochondria, Oxidative Stress and Muscle Protection”, Unistra, Faculty of Medicine, Translational Medicine Federation of Strasbourg (FMTS), University of Strasbourg, 11 rue Humann, 67000 Strasbourg, France; marianne.riou@chru-strasbourg.fr (M.R.); walid.oulehri@chru-strasbourg.fr (W.O.); olivier.rouyer@chru-strasbourg.fr (O.R.); alain.meyer1@chru-strasbourg.fr (A.M.); irina.enache@chru-strasbourg.fr (I.E.); cristina.pistea@chru-strasbourg.fr (C.P.); anne.charloux@chru-strasbourg.fr (A.C.); Frederic.deblay@chru-strasbourg.fr (F.d.B.); olivier.collange@chru-strasbourg.fr (O.C.); michel.mertes@chru-strasbourg.fr (M.M.); emmanuel.andres@chru-strasbourg.fr (E.A.); samy.talha@chru-strasbourg.fr (S.T.); 2 Physiology and Functional Exploration Service, University Hospital of Strasbourg, CHU, 1 Place de l’hôpital, 67091 Strasbourg, France; cedric.momas@chru-strasbourg.fr (C.M.); Fabienne.lebourg@chru-strasbourg.fr (F.L.); 3 Department of Anesthesiology and Surgical Critical Care, University Hospital of Strasbourg, 1 Place de l’hôpital, 67091 Strasbourg, France; 4 Department of Pneumology, University Hospital of Strasbourg, 1 Place de l’hôpital, 67091 Strasbourg, France; christophe.marcot@chru-strasbourg.fr; 5 Department of Internal Medicine, University Hospital of Strasbourg, 1 Place de l’hôpital, 67091 Strasbourg, France

**Keywords:** COVID-19, vascular function, flow-mediated dilatation

## Abstract

The coronavirus disease 2019 (COVID-19) pandemic has spread rapidly worldwide, with more than two million deaths. Evidence indicates the critical role of the vascular endothelium in its pathophysiology but, like potential changes in functional vasodilation, the vascular effect of SARS-CoV-2 at a given distance from the acute infection is largely unknown. We assessed brachial artery flow-mediated dilatation (FMD) in 27 COVID-19 patients needing conventional or intensive care unit hospitalization, three months after SARS-CoV-2 infection diagnosis and in nine age- and sex- matched control subjects. Interestingly, the FMD was lower in COVID-19 patients as compared to controls (8.2 (7.2–8.9) vs. 10.3 (9.1–11.7)); *p* = 0.002, and half of the hospitalized COVID-19 survivors presented with a reduced FMD < 8% at three months of COVID-19 onset. Impaired FMD was not associated with severe or critical SARS-CoV-2 infection, reflected by ICU hospitalization, total hospitalization duration, or severity of lung damage. In conclusion, reduced FMD is often observed even three months after hospitalization for SARS-CoV-2 infection, but such alteration predominantly appears to not be related to COVID-19 severity. Longer and larger follow-up studies will help to clarify the potential prognosis value of FMD among COVID-19 patients, as well as to further determine the mechanisms involved.

## 1. Introduction

The coronavirus disease 2019 (COVID-19) pandemic, caused by severe acute respiratory syndrome coronavirus 2 (SARS-CoV-2), has spread worldwide rapidly, with several million individuals infected and more than two million deaths [1]. Evidence indicates the critical role of the vascular endothelium in the pathophysiology of COVID-19 [2]. Inflammation and a pro-coagulability state described in COVID-19 cases are both a cause and consequence of endothelial cell (ECs) dysfunction, named “endotheliitis” [3]. Indeed, electron microscopy studies have reported SARS-CoV-2 viral particles within the endothelium of different organs, suggesting that endothelial cell injury or activation could be a central feature of the COVID-19-associated-coagulopathy, particularly during the inflammation phase of the disease [3]. Moreover, SARS-CoV-2 binds to the transmembrane angiotensin-converting enzyme 2 (ACE 2) receptors that are abundant in the vasculature and, notably, in vascular ECs.

However, as with potential changes in functional vasodilation, the vascular effect of SARS-CoV-2 at distance of the acute infection is largely unknown. Recently, it has been proposed that endothelial biomarkers and test of function (using the flow-mediated dilation (FMD) technique) should be evaluated for their value, both as risk stratification and in the early detection of vascular sequelae, as well as for long-term cardiovascular complications in COVID-19 patients [4]. Indeed, FMD of the brachial artery is a noninvasive measure of vasomotor function that determines arterial dilatation in response to a shear stress stimulus, producing a nitric oxide (NO)-dependent response. Here, FMD reflects endothelial dysfunction, which predicts long-term cardiovascular events [5,6]. 

The aims of this work were, therefore, to determine, for the first time, whether FMD might be reduced in severe COVID-19 survivors three months after infection, and whether COVID-19 severity per se might influence the patients’ FMD value.

## 2. Population and Methods

### 2.1. Patients

We assessed brachial artery FMD in 27 COVID-19 patients hospitalized in Strasbourg University Hospital, France during the first outbreak of COVID-19 (March–April 2020), three months after SARS-CoV-2 infection diagnosis was confirmed by a nasopharyngeal swab polymerase chain reaction. Patients were stratified in two groups according to the severity of COVID-19 during hospitalization, as defined by the World Health Organization (WHO) [7], Table 1: mild to moderate (non-severe) diseased, and severe or critical diseased patients (defined by the criteria of acute respiratory distress syndrome (ARDS), sepsis, septic shock, or other conditions that would normally require the provision of life-sustaining therapies such as mechanical ventilation and/or clinical signs of severe respiratory distress during hospitalization). These patients were compared to a control group (*n* = 9) without COVID history and of a similar age—59 years old (interquartile range (IQR), 54–62), as well as gender (56% male).

All subjects provided informed consent, and the study was approved by the local ethic committee (CE-2020-193).

### 2.2. Methods

After a 20-min rest, scans were recorded over one minute to assess baseline vessel diameter. Then, ischemia was induced for five minutes with a blood pressure cuff placed around the forearm at 10 cm, distal to the brachial artery segment and inflated to a supra systolic pressure of 200 mmHg. After cuff deflation, the reactive hyperemia increased brachial artery diameter, and the FMD was recorded until three minutes thereafter. During the procedure, participants were continuously monitored with an electrocardiogram lead, where systolic and diastolic brachial pressures were obtained.

The FMD was determined by the same trained observer in all subjects, and expressed as the maximal percentage change in post-stimulus vessel diameter. This was calculated as follows: FMD (%) = [(Max diameter (mm) − Resting diameter (mm))/Resting diameter (mm)] × 100. In our team, the normal range of FMD values is around 10 ± 1% [8,9], with FMD being considered more predictive of cardiovascular complications when inferior to 8%. Thus, for instance, impaired brachial-artery endothelial function independently predicted long-term cardiovascular events in patients with peripheral arterial disease, with the risk being approximately nine-fold higher in patients with FMD < 8.1% [10]. Furthermore, an FMD of < 8% can predict patients with occult coronary plaques whose conventional coronary angiographies revealed normal coronary arteries [11].

To investigate whether COVID-19 severity might influence the patients’ FMD per se, we analyzed the characteristics of hospitalization and chest computer tomography (CT) scans, as well as the main factors reported to modulate FMD.

### 2.3. Statistical Analysis

Descriptive analyses of quantitative data comprised the median and dispersion parameters. Qualitative data were described according to population sizes and percentages. The Charlson Comorbidity Index Score was calculated for each patient. First, we compared all COVID-19 patients to the control group. Then, comparisons between two groups of COVID-19 patients were performed according to disease severity. Comparisons between groups were conducted using the chi-squared test for percentages, and the *t*-test for continuous variables. *p* < 0.05 was considered statistically significant.

## 3. Results

### 3.1. Overall Population

As reported in Table 2, in the 27 patients the median value of brachial artery FMD was 8.1% (IQR, 7.2–8.9), whilst 12 patients (44%) had an FMD of <8%. 

When considering the entire population, FMD was lower in COVID-19 patients as compared to controls (8.2 (7.2–8.9) vs. 10.3 (9.11–11.7), respectively; *p* = 0.002).

**Figure 1:**
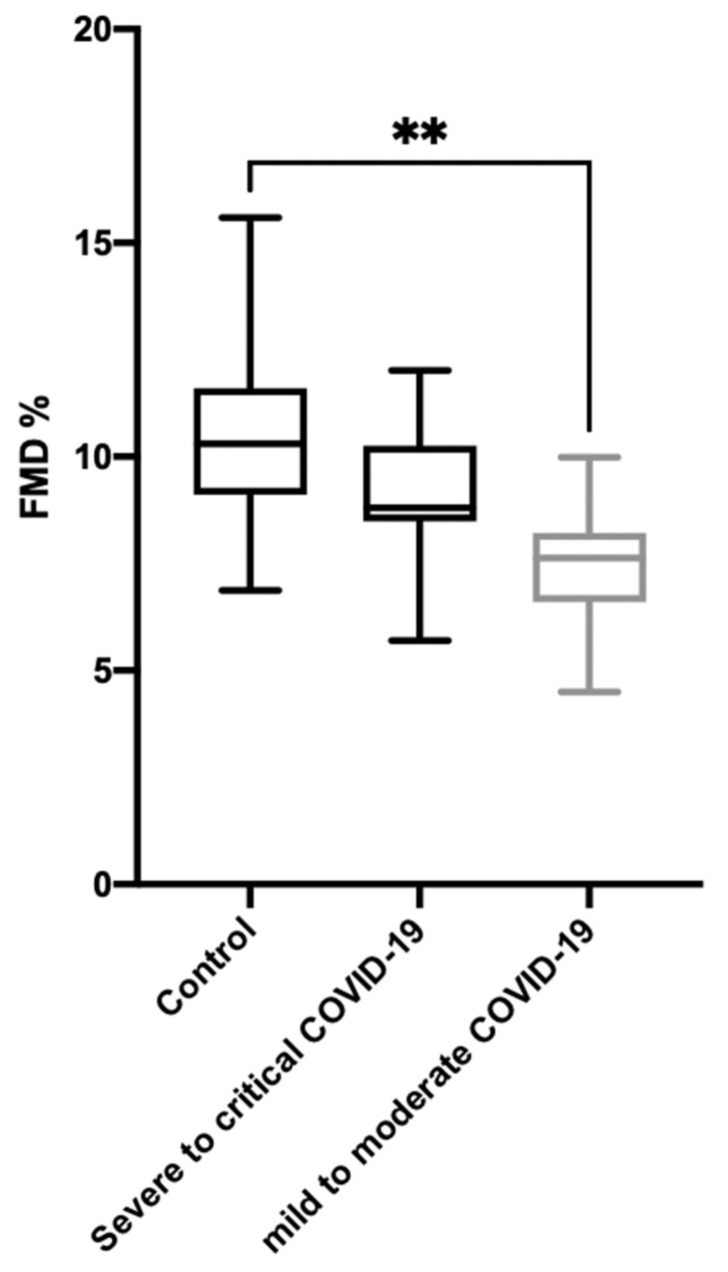
Distribution of flow-mediated dilation (FMD) in controls and COVID-19 patients, classified following the WHO guidelines. ** *p* < 0.001.

More precisely, using the WHO classification for COVID-19 patients, the 16 mild-to-moderate infected patients had a median brachial artery FMD value of 7.6% (IQR, 6.6–8.2; *p* = 0.0011 as compared to controls), and the 11 severe-to-critical diseased patients had a median brachial artery FMD value of 8.8% (IQR, 8.5–10.2; *p* = 0.098 as compared to controls and *p* = 0.016 as compared to mild to moderate patients); see Figure 1.

The patients’ median age was 57 years old (IQR, 49–66), and 63% were male. Median body mass index was 29 kg·m^−2^ (IQR, 26.2–34). Systemic blood pressures were 134/84 mmHg for systolic and diastolic pressures, respectively. The most common comorbidities were hypertension (48%), diabetes mellitus (26%), and sleep apnea syndrome (22%). Twenty-two percent of the patients did smoke, and the Charlson Comorbidity Index score was 2; Table 2.

Regarding COVID-19 severity, all patients were hospitalized, and the median hospitalization duration was 14 days (IQR, 8–42). As defined by the WHO, COVID-19 patients were stratified in two groups: mild-to-moderate disease (*n* = 16), and severe-to-critical disease (*n* = 11) [12]. Forty-one percent of the patients (*n* = 11) needed hospitalization in intensive care units (ICU) for acute respiratory distress syndrome. Besides hospitalization characteristics, the severity of COVID-19 was also determined using chest CT scans upon admission. As reported in the literature, severe/critical stage observed when >50% of the lung parenchyma was abnormal [11], was present in one-third of the patients. Pulmonary embolism was observed in 15% of the patients.

### 3.2. Comparison between Mild-to-Moderate and Severe or Critical Diseased Patients

Few, but interesting, differences were observed between the two groups of patients. The patients with mild-to-moderate disease had a slight tendency to be older (57 vs. 51 years old), but, like other clinical characteristics, this failed to reach statistical significance (*p* = 0.84). They were hospitalized for a shorter time (12 vs. 42 days, *p* < 0.001) than patients with severe-to-critical disease, and they were not hospitalized in an ICU (Table 3). No other significant differences were observed regarding other comorbidities between the two groups of patients.

## 4. Discussion and Conclusions

The main result of this study is that almost half of the hospitalized COVID-19 survivors presented with an FMD of <8% at three months of COVID-19 onset. In particular, the 16 mild-to-moderate COVID-19 patients had a significantly decreased FMD as compared to the controls, whereas the FMD only tended to be lower in the severe-to-critical COVID-19 patients. Unexpectedly, the lower FMD was not observed in patients with severe or critical COVID-19 infection, reflected by ICU hospitalization, total hospitalization duration, and severity of lung damage on chest CT scans performed upon admission.

To date, there is only one report published on FMD measures in patients suffering from COVID-19. Ratchford et al. recently showed a significant and striking reduction in brachial artery FMD among 11 young COVID-19 patients relatively early (3–4 weeks) following the disease onset [13]. In this interesting study, reactive hyperemia response to forearm cuff occlusion was not modified in COVID-19 patients, suggesting a central role of NO in the vascular dysfunction observed after acute infection. Thus, although no long-term and longitudinal data, or FMD values before COVID-19 onset, were available, this study supports the possibility that COVID-19 has deleterious effects on vascular dilatation capacity.

Our data, obtained in older and more severe COVID-19 patients at three months following the disease’s onset, extend existing knowledge on vascular function in COVID-19 patients. We confirm that the FMD can be reduced in such patients; however, a significant percentage of them did not demonstrate an impaired vasodilatory capacity. Whether an initial impairment may have been improved later on, together with a better NO availability or efficacy, cannot be inferred from our data [14]. Indeed, as in the previous study, a causal relationship between vascular impairment and the virus is not possible to demonstrate clearly, since FMD was not determined before SARS-CoV-2 infection. The decreased FMD observed in these patients may also be related to other parameters, possibly including pre-existing comorbidities. In particular, aging is independently associated with progressive endothelial dysfunction and impaired FMD in humans [15]. Here, patients with reduced FMD tended to be older than patients with an FMD of ≥ 8%. Alternatively, other factors known to impair FMD may have played a role. We therefore investigated a potential link between inflammatory markers during hospitalization, Tocilizumab, as well as drugs modulating blood pressure, lipid lowering drugs, and the FMD. None appeared significant, and a larger population may be needed. During hospitalization, no difference was observed regarding CRP values in our patients, and no correlation was found between the CRP during the acute phase and FMD values three months later.

There are several limitations in this study. Firstly, results were obtained via a relatively small sample size of COVID-19 survivors, and further studies using a larger population will be useful to investigate whether the FMD decrease would be greater in subjects with major cardiovascular risk factors, and whether FMD might have prognostic value in such settings. Secondly, although almost impossible to obtain, no data on FMD values before infection were available, despite the fact that this may have been helpful to evaluate the specific impact of SARS-CoV-2 infection on FMD values. Ideally, a longitudinal study investigating patients’ FMD prior to COVID-19 infection, and again later on would provide interesting data, but may prove impossible to perform.

To conclude, our data support that reduced FMD can be observed in almost half of middle-aged patients three months after hospitalization for SARS-CoV-2 infection, and, that this appears to not be mainly related to COVID-19 severity. Longer and larger follow-up studies will be helpful to clarify the potential prognostic value of FMD among COVID-19 survivors, and to further determine the mechanisms involved.

## Figures and Tables

**Table 1:** WHO severity definitions of COVID-19 infection [7].

Critical COVID-19: Defined by the criteria for acute respiratory distress syndrome (ARDS), sepsis, septic shock, or other conditions that would normally require the provision of life-sustaining therapies such as mechanical ventilation (invasive or non-invasive) or vasopressor therapy.
Severe COVID-19: Defined by any of the following: o Oxygen saturation <90% on room air; O Respiratory rate >30 breaths/min in adults; o Signs of severe respiratory distress (accessory muscle use, inability to complete full sentences, and, in children, very severe chest wall indrawing, grunting, central cyanosis, or presence of any other general danger signs).
Non-severe (mild-to-moderate) COVID-19: Defined as absence of any criteria for severe or critical COVID-19.

**Table 2:** Clinical characteristics of the 27 patients, 3 months after COVID-19.

	Total Population (*n* = 27)	Mild to Moderate Disease (*n* = 16)	Severe to Critical Disease (*n* = 11)	*p*
FMD (%)	8.1 (7.2–8.9)	7.6 (6.6–8.2)	8.8 (8.5–10.2)	0.016
Clinical characteristics				
Age (years)	57 (49–66)	57 (51–66)	51 (46–73)	0.84
Male (*n*/%)	17/63%	9 (56%)	8 (73%)	0.38
BMI (Kg/m^2^)	29 (26.2–34)	29.5 (26.4–34.1)	27.1 (25.6–32.1)	0.33
Systolic blood pressure (mmHg)	134 (128–140)	137 (129–153)	132 (124–137)	0.12
Diastolic blood pressure (mmHg)	84 (77–93)	85 (81–98)	78 (75–91)	0.19
Hypertension (*n*/%)	13/48%	6/37%	7/64%	0.18
Diabetes (*n*/%)	7/26%	5/31%	2/18%	0.44
Sleep apnea syndrome (*n*/%)	6/22%	3/19%	3/27%	0.60
Chronic heart failure (*n*/%)	0	0	0	-
Former or active smokers (*n*/%)	6/22%	4/25%	2/18%	0.68
Charlson Comorbidity Index Score	2 (0–3)	2 (1–3)	2 (0–4)	0.91

BMI: body mass index; FMD: flow-mediated dilation; ICU: intensive care unit. CT: chest computerized tomography. Descriptive analyses of quantitative data comprise the median values and interquartile ranges. Qualitative data are described according to population sizes and percentages. *p* < 0.05 is considered statistically significant.

**Table 3:** Hospitalization characteristics of the 27 patients, 3 months after COVID-19.

	Total Population (*n* = 27)	Mild-to-Moderate Disease (*n* = 16)	Severe-to-Critical Disease (*n* = 11)	*p*
FMD (%)	8.1 (7.2–8.9)	7.6 (6.6–8.2)	8.8 (8.5–10.2)	0.016
Hospitalization characteristics				
Total hospitalization duration (days)	14 (8–42)	12 (7–15)	42 (21–53)	<0.001
ICU hospitalization (Yes, *n*/%)	11/41%	0	11/100%	<0.001
Chest CT on admission with severe/critical (>50%) abnormal lung parenchyma (Yes, *n*/%)	9/33%	0	8/73%	<0.001
Pulmonary embolism during hospitalization	4/15%	0	4/36%	0.009

ICU: intensive care unit. CT: chest computerized tomography. Descriptive analyses of quantitative data comprise the median values and interquartile ranges. Qualitative data is described according to population sizes and percentages. *p* < 0.05 is considered statistically significant. *n*/%: number and percentage of patients needing ICU hospitalization or with (>50%) abnormal lung parenchyma on admission chest CT.

## Data Availability

The data presented in this study are available on request from the corresponding author. The data are not publicly available due to privacy and ethical reasons.

## References

[1] Zhu N., Zhang D., Wang W., Li X., Yang B., Song J., Zhao X., Huang B., Shi W., Lu R. (2020). A Novel Coronavirus from Patients with Pneumonia in China, 2019. N. Engl. J. Med..

[2] Ackermann M., Verleden S.E., Kuehnel M., Haverich A., Welte T., Laenger F., Vanstapel A., Werlein C., Stark H., Tzankov A. (2020). Pulmonary Vascular Endothelialitis, Thrombosis, and Angiogenesis in Covid-19. N. Engl. J. Med..

[3] Varga Z., Flammer A.J., Steiger P., Haberecker M., Andermatt R., Zinkernagel A.S., Mehra M.R., Schuepbach R.A., Ruschitzka F., Moch H. (2020). Endothelial Cell Infection and Endotheliitis in COVID-19. Lancet.

[4] Evans P.C., Ed Rainger G., Mason J.C., Guzik T.J., Osto E., Stamataki Z., Neil D., Hoefer I.E., Fragiadaki M., Waltenberger J. (2020). Endothelial Dysfunction in COVID-19: A Position Paper of the ESC Working Group for Atherosclerosis and Vascular Biology, and the ESC Council of Basic Cardiovascular Science. Cardiovasc. Res..

[5] Thijssen D.H.J., Bruno R.M., van Mil A.C.C.M., Holder S.M., Faita F., Greyling A., Zock P.L., Taddei S., Deanfield J.E., Luscher T. (2019). Expert Consensus and Evidence-Based Recommendations for the Assessment of Flow-Mediated Dilation in Humans. Eur. Heart J..

[6] Inaba Y., Chen J.A., Bergmann S.R. (2010). Prediction of future cardiovascular outcomes by flow-mediated vasodilatation of brachial artery: A metaanalysis. Int. J. Cardiovasc. Imaging.

[7] WHO. https://www.Who.Int/Publications/i/Item/Clinical-Management-of-Covid-19.

[8] Rouyer O., Auger C., Charles A.-L., Talha S., Meyer A., Piquard F., Andres E., Schini-Kerth V., Geny B. (2019). Effects of a High Fat Meal Associated with Water, Juice, or Champagne Consumption on Endothelial Function and Markers of Oxidative Stress and Inflammation in Young, Healthy Subjects. J. Clin. Med..

[9] Rouyer O., Talha S., Di Marco P., Ellero B., Doutreleau S., Diemunsch P., Piquard F., Geny B. (2009). Lack of endothelial dysfunction in patients under tacrolimus after orthotopic liver transplantation. Clin. Transplant..

[10] Gokce N., Keaney J.F.J., Hunter L.M., Watkins M.T., Nedeljkovic Z.S., Menzoian J.O., Vita J.A. (2003). Predictive Value of Noninvasively Determined Endothelial Dysfunction for Long-Term Cardiovascular Events in Patients with Peripheral Vascular Disease. J. Am. Coll. Cardiol..

[11] Mutlu B., Tigen K., Gurel E., Ozben B., Karaahmet T., Basaran Y. (2011). The Predictive Value of Flow-Mediated Dilation and Carotid Artery Intima-Media Thickness for Occult Coronary Artery Disease. Echocardiography.

[12] Revel M.-P., Parkar A.P., Prosch H., Silva M., Sverzellati N., Gleeson F., Brady A. (2020). COVID-19 Patients and the Radiology Department—Advice from the European Society of Radiology (ESR) and the European Society of Thoracic Imaging (ESTI). Eur. Radiol..

[13] Ratchford S.M., Stickford J.L., Province V.M., Stute N., Augenreich M.A., Koontz L.K., Bobo L.K., Stickford A.S.L. (2020). Vascular Alterations Among Young Adults with SARS-CoV-2. Am. J. Physiol. Heart Circ. Physiol..

[14] Green S.J. (2020). Covid-19 Accelerates Endothelial Dysfunction and Nitric Oxide Deficiency. Microbes Infect..

[15] Celermajer D.S., Sorensen K.E., Spiegelhalter D.J., Georgakopoulos D., Robinson J., Deanfield J.E. (1994). Aging Is Associated with Endothelial Dysfunction in Healthy Men Years before the Age-Related Decline in Women. J. Am. Coll. Cardiol..

